# Supplementary material for: Single-domain antibody delivery using an mRNA platform protects against lethal doses of botulinum neurotoxin A
Source: Front Immunol. 2023 Feb 14;14:1098302. doi: 10.3389/fimmu.2023.1098302 (PMC9971915; doi:10.3389/fimmu.2023.1098302)
Supplement: Supplementary Figure 1 — The influence of the presence of a cap1/cap0 structure and incorporation of modified nucleoside, N1-methylpseudouridine (m1Ψ), on the firefly luciferase mRNA expression in mouse bone marrow-derived dendritic cells (mBMDC). [file Image_1.pdf]

SUPPLEMENTARY MATERIAL

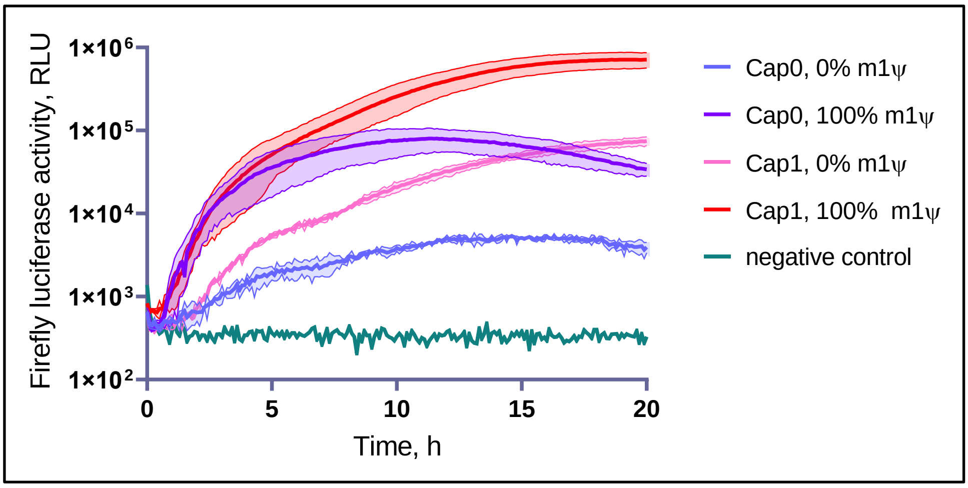

Figure S1. The influence of the presence of a cap1/cap0 structure and incorporation of modified nucleoside, N1-methylpseudouridine ($m^1\Psi$), on the firefly luciferase mRNA expression in mouse bone marrow-derived dendritic cells (mBMDC).
