# Supplementary material for: Single-domain antibody delivery using an mRNA platform protects against lethal doses of botulinum neurotoxin A
Source: Front Immunol. 2023 Feb 14;14:1098302. doi: 10.3389/fimmu.2023.1098302 (PMC9971915; doi:10.3389/fimmu.2023.1098302)
Supplement: Supplementary Figure 2 — Representative IVIS images of BALB/c mice injected with 5.0 µg mRNA-LNP in PBS (right and middle mouse), or PBS (left mouse) by intramuscular (i.m.) route. Relative luminescence plot is shown, and the scale of luminescence is indicated. [file Image_2.pdf]

SUPPLEMENTARY MATERIAL

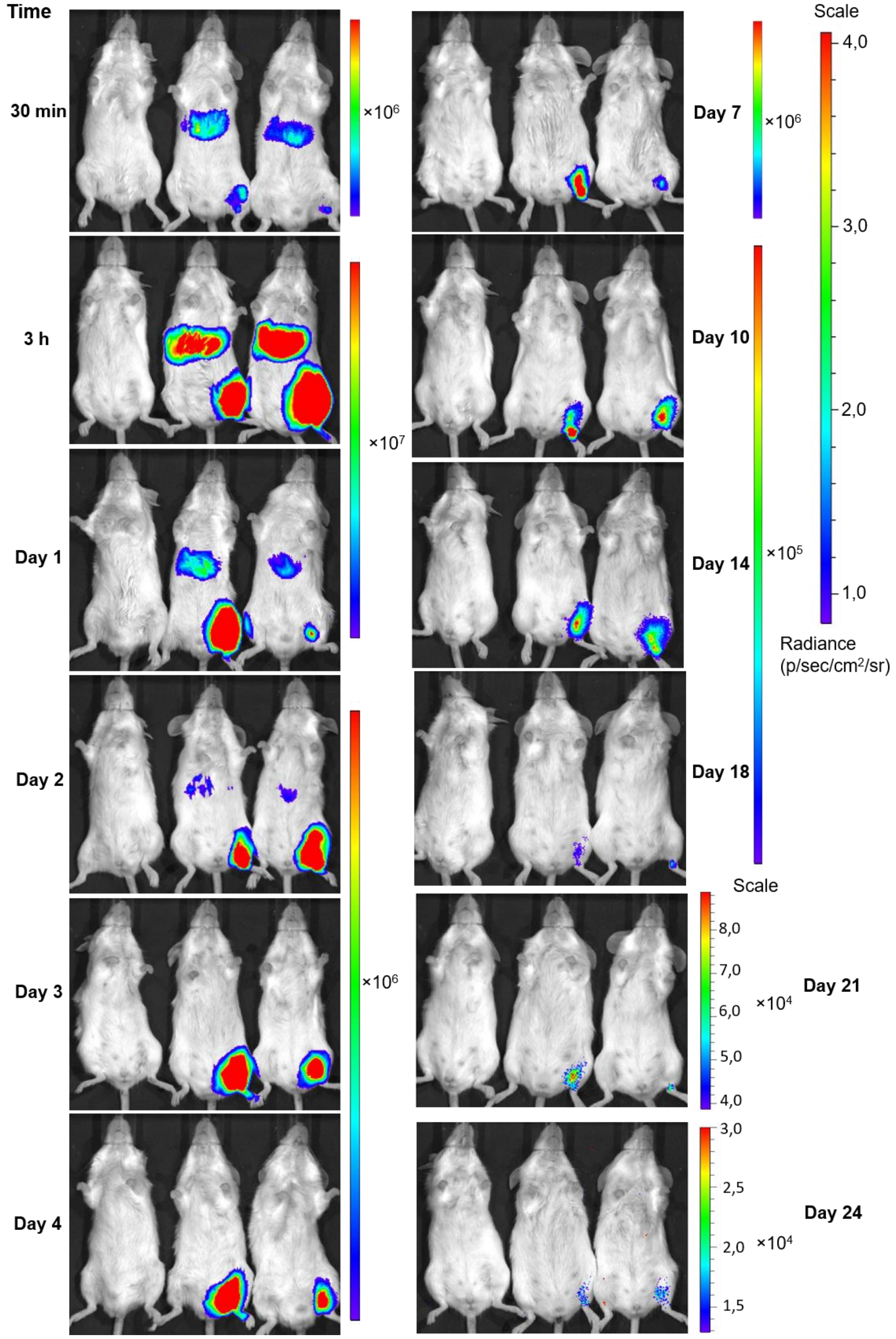

Figure S2. Representative IVIS images of BALB/c mice injected with 5.0 µg mRNA-LNP in PBS (right and middle mouse), or PBS (left mouse) by intramuscular (i.m.) route. Relative luminescence plot is shown, and the scale of luminescence is indicated.
