# Supplementary material for: Single-domain antibody delivery using an mRNA platform protects against lethal doses of botulinum neurotoxin A
Source: Front Immunol. 2023 Feb 14;14:1098302. doi: 10.3389/fimmu.2023.1098302 (PMC9971915; doi:10.3389/fimmu.2023.1098302)
Supplement: Supplementary Figure 3 — Cloning strategy for IVT plasmid templates based on pJAZZ-OK (Details described in Methods section 1). (A). Schematic of commercial pJAZZ-OK linear bacterial plasmid derived from N15 bacteriophage genome (telN – protelomerase gene, repA – replication factor gene and origin of replication, cB – the primary replication repressor protein, Kan R – kanamycin resistance gene, black semi-circles denoting terminal hairpin loops); (B). Schematic of linear plasmid with ColE1 origin of replication (ori, yellow arrow) followed by SmaI restriction site (grey block); (C) Schematic of plasmid contained backbone sequences for IVT: T7-promoter (T7P, white triangle), human HBA1 5′ and 3′ UTRs (blue and plum blocks, respectively) cleaved by SmaI site, 2x strong translation terminator (Stop, red octagon), 100-nt poly(A)-tail (orange block) followed by BsmBI restriction site (cyan rhombus); (D) Schematic of resulted linear plasmid with open reading frame of luciferase or B11 antibody (GOI ORF, green arrow). [file Image_3.pdf]

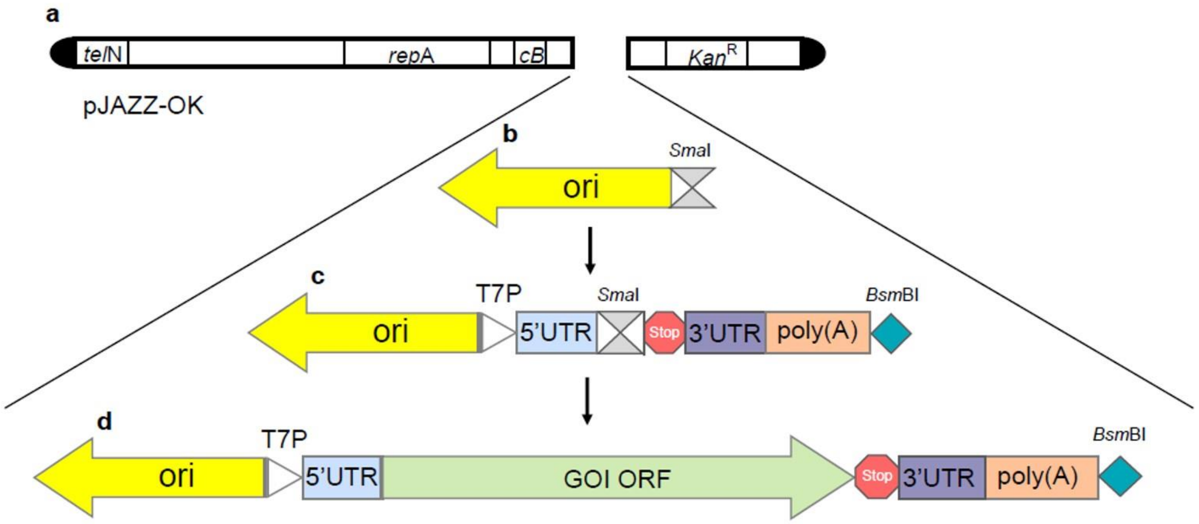

Figure S3. Cloning strategy for IVT plasmid templates based on pJAZZ-OK (Details described in Methods section 1). **a.** Schematic of commercial pJAZZ-OK linear bacterial plasmid derived from N15 bacteriophage genome (*telN* – protelomerase gene, *rep*A – replication factor gene and origin of replication, *cB* – the primary replication repressor protein, Kan^R – kanamycin resistance gene, black semi-circles denoting terminal hairpin loops); **b.** Schematic of linear plasmid with *Col*E1 origin of replication (*ori*, yellow arrow) followed by *Sma*I restriction site (grey block); **c.** Schematic of plasmid contained backbone sequences for IVT: T7-promoter (*T7P*, white triangle), human *HBA1* 5′ and 3′ UTRs (blue and plum blocks, respectively) cleaved by *Sma*I site, 2x strong translation terminator (*Stop*, red octagon), 100-nt poly(A)-tail (orange block) followed by *BsmB*I restriction site (cyan rhombus); **d.** Schematic of resulted linear plasmid with open reading frame of luciferase or B11 antibody (*GOI ORF*, green arrow).
