# Supplementary material for: Single-domain antibody delivery using an mRNA platform protects against lethal doses of botulinum neurotoxin A
Source: Front Immunol. 2023 Feb 14;14:1098302. doi: 10.3389/fimmu.2023.1098302 (PMC9971915; doi:10.3389/fimmu.2023.1098302)
Supplement: Supplementary Table 1 — The primer sets used in the study. [file Table_1.docx]

**SUPPLEMENTARY MATERIAL**

**Table S1.** **The primer sets used in the study**

| **Primer name** | **Sequence (5’ to 3’)** | **Description** |
| --- | --- | --- |
| OriF | cgggTTGAGATCCTTTTTTTCTGC | *Col*E1replication origin insertion into pJAZZ-OK |
| OriR | cgtacgTTTCCATAGGCTCCGC |  |
| T7PF | tttCCACCTGACaattcTAATACGACTCACTATA | Cloning of mRNA template elements |
| 5'Sma3'R | AGGCTCCAGCTTATATTTAgagacccGGGTTCTCTCTGAGTCTGTGGGGACCAGAAGAAT |  |
| Sma3'F | gggtctcTAAATATAAGCTGGAGCCTCG |  |
| 3'UTRR | TGCTGGCCTTTTGCTCcgtctcATTT |  |
| 5'LucF | TCTGGTCCCCACAGACTCAGAGAGAACCCGCCACCATGGAAGACGCCAAAAACATTAAGAagggc | FLuc CDS insertion between 5’ and 3’UTRs |
| 3'LucR | GGGCAAGAAGCTAGGCCACCGAGGCTCCAGCTTATATTTAgagaccCACGGCGATCTTGCCGCC |  |
| B11-EspF | Ctcaggatcctcgtgagaaacgtccacaacgaca | *Bsm*BI site mutagenesis in B11-Fc CDS |
| B11F | CACAGACTCAGAGAGAACCCGCCACCatgggctggagtctgatccttctg | B11-Fc CDS insertion between 5’ and 3’UTRs |
| B11R | GCCACCGAGGCTCCAGCTTATATTTAtcacttgccaggagacaaagacagg |  |
| SL1 | CAGTCCAGTTACGCTGGAGTC | Sanger Sequencing |
| OriSeqF | GGCGGTGCTACAGAGTT |  |
| NZ RevC | AAATGGTCAGTTAATCAGTTCT |  |
| 3'UTRF | CTCGGTGGCCTAGCTTCTTGCCCCTT |  |
| LucF1seq | ACATATCGAGGTGGACATTAC |  |
| LucF2seq | CGACACCGCTATCCTCAG |  |
| LucF3seq | TACTGGGACGAGGACGAG |  |
| B11seqF | CTGTTTCCTCCCAAACCTAA |  |
